# Heteroleptic Metal Complexes of 4‐(2‐Pyridylazo)resorcinol and 1,10‐Phenanthroline: Synthesis, Comprehensive Characterization, Electrochemical and Antioxidant Properties, and Computational Investigations

**DOI:** 10.1155/bca/6848871

**Published:** 2026-08-03

**Authors:** Fatma Karipcin, Neşet Özdemir, Bilge Bıçak, Gizem Yıldırım Baştemur, Reyhan Akpinar, Serda Kecel Gündüz, Sabriye Percin Ozkorucuklu

**Affiliations:** ^1^ Department of Chemistry, Faculty of Arts and Sciences, Nevşehir Hacı Bektaş Veli University, Nevşehir, Türkiye; ^2^ Department of Physics, Faculty of Sciences, Istanbul University, Istanbul, Türkiye, istanbul.edu.tr; ^3^ Department of Molecular Biology and Genetics, Faculty of Sciences, Istanbul University, İstanbul, Türkiye, istanbul.edu.tr; ^4^ Programme of Molecular Biotechnology and Genetics, Institute of Graduate Studies in Science Istanbul University, Istanbul, Türkiye; ^5^ Center for Research and Practice in Biotechnology and Genetic Engineering, Istanbul University, Istanbul, Türkiye, istanbul.edu.tr

**Keywords:** 4-(2-pyridylazo)resorcinol, antioxidant activity, electrochemical behavior, heteroligand complexes, molecular docking, phenanthroline

## Abstract

New heteroligand metal complexes, [M(phen) (PAR) (OAc)]·H_2_O (M = Mn(II) (1), Cu(II) (3)) and [Co(phen) (PAR) (H_2_O)]OAc·H_2_O (2), incorporating 4‐(2‐pyridylazo)resorcinol (PAR) as the primary ligand and 1,10‐phenanthroline (phen) as the secondary ligand, were synthesized and comprehensively characterized. Elemental analysis, FT‐IR spectroscopy, thermal analysis, magnetic susceptibility measurements, and molar conductivity studies confirmed the proposed compositions and coordination modes of the complexes. Spectroscopic data revealed that PAR coordinates to the metal center in its deprotonated form through the resorcinol oxygen atom and the azo and pyridyl nitrogen atoms, while phen acts as a bidentate N,N‐donor ligand. The electrochemical properties of the complexes were investigated by cyclic voltammetry using a glassy carbon electrode in DMF containing 0.1 M TBAP as the supporting electrolyte. All complexes exhibited well‐defined redox behavior governed predominantly by diffusion‐controlled electrochemical processes. The antioxidant activities of the synthesized complexes were evaluated using 2,2‐diphenyl‐1‐picrylhydrazyl (DPPH) radical‐scavenging and cupric ion reducing antioxidant capacity (CUPRAC) assays, revealing notable antioxidant responses in both methods. Density functional theory (DFT) calculations, including HOMO‐LUMO and molecular electrostatic potential (MEP) analyses, provided insights into the electronic structures and reactive sites of the complexes. Furthermore, molecular docking studies demonstrated favorable binding affinities toward xanthine oxidase (XO) and Keap1 receptors. The stability of the predicted protein–ligand interactions was further confirmed by 100 ns molecular dynamics simulations, which revealed stable trajectories, compact binding modes, and persistent intermolecular interactions throughout the simulation period.

## 1. Introduction

Azo compounds are an important class of organic molecules widely used as ligands in coordination chemistry due to their strong chelating ability and structural versatility. Their ability to coordinate metal ions through azo nitrogen and additional donor atoms makes them suitable for the construction of stable metal complexes with tunable electronic and physicochemical properties. In addition, azo‐based ligands can influence the redox behavior and biological activity of metal centers, making them attractive for functional material and bioinorganic chemistry studies [1–3].

Among azo ligands, 4‐(2‐pyridylazo)resorcinol (PAR) is a well‐known tridentate chelator capable of coordinating metal ions through phenolic oxygen, azo nitrogen, and pyridyl nitrogen atoms, forming stable complexes over a wide pH range. Similarly, 1,10‐phenanthroline (phen), a rigid N,N‐donor heterocyclic ligand, is frequently employed in mixed‐ligand systems due to its strong metal‐binding ability and its influence on the electronic and biological properties of metal complexes [1–5]. Many azo‐metal complexes show significant antimicrobial properties, with the metal ions playing a crucial role in the mechanism of action. These complexes can interact with microbial cell walls, inhibiting growth or inducing cell death, thereby making them potential candidates for antimicrobial drug development [6–11]. Some studies have highlighted the anticancer activity of azo‐metal complexes, which can induce cytotoxic effects in cancer cells. The metal center may facilitate the complex’s ability to bind to and interact with DNA, thereby disrupting cell division and inducing apoptosis [10, 12, 13]. Metal coordination in azo ligands can also impart antioxidant properties, helping to neutralize free radicals in biological systems. This makes azo‐based complexes potential candidates for use in mitigating oxidative stress–related diseases, such as neurodegenerative disorders [14, 15]. Azo ligands are also used in analytical chemistry due to their ability to form colored complexes with various metal ions, which can be quantified spectrophotometrically. This property makes them useful in trace metal analysis and environmental monitoring. Azo ligands and their metal complexes exhibit a wide range of chemical and biological properties, making them important in both scientific research and practical applications, especially in fields, such as pharmacology, environmental science, and materials chemistry [2]. In this study, PAR, a commercial azo dye that can coordinate metal ions with its pyridine nitrogen atom, azo nitrogen, and ortho‐hydroxyl group oxygen, was selected as the primary ligand. PAR is a chelating ligand capable of forming stable complexes with various transition and heavy metals over a wide pH range [16–18].

1,10‐Phenanthroline (phen), a well‐known N,N‐heterocyclic ligand, is widely used as a secondary ligand in mixed‐ligand complexes because it possesses a delocalized π‐electron system, readily forms stable chelates with a wide range of metal ions, and its complexes exhibit diverse biological functions and pharmacological activities. In addition, 1,10‐phenanthroline significantly influences the toxicity of the metal ion to which it is coordinated [19–24].

Mixed‐ligand metal complexes have attracted considerable attention because the combination of different ligands around a metal center allows fine‐tuning of structural, electrochemical, and biological properties. Such systems often exhibit enhanced stability and improved functional behavior compared to their single‐ligand counterparts, making them of interest in catalysis, sensing, and medicinal chemistry applications [20, 25, 26].

Transition metal ions, such as Mn(II), Co(II), and Cu(II), are particularly suitable for the construction of such systems due to their variable coordination behavior, accessible redox states, and biological relevance. Mn(II) is associated with antioxidant enzymatic systems, Co(II) displays flexible coordination environments, and Cu(II) is well known for its rich redox chemistry and biological activity. Therefore, comparative studies of these metal ions can provide insight into structure–property relationships in coordination compounds [27–29].

Although phenanthroline‐ and PAR‐based metal complexes have been previously reported, the combination of these ligands with Mn(II), Co(II), and Cu(II) in mixed‐ligand systems and their systematic investigation in terms of electrochemical behavior, antioxidant activity, and theoretical electronic structure remains limited.

In this study, we report the synthesis, characterization, electrochemical behavior, antioxidant activity, and density functional theory (DFT) calculations of new mixed‐ligand metal(II) complexes, [M(phen) (PAR)X]Y (M = Mn(II), Co(II), Cu(II)). The present work aims to explore the effect of metal ion variation on the structural, electrochemical, and antioxidant properties of the complexes and to provide a comprehensive understanding of their structure–property relationships.

## 2. Materials and Methods

### 2.1. Reagents and Chemicals

All reagents and solvents used were of analytical grade and employed without further purification. 2,4,6‐Tris(2‐pyridyl)‐s‐triazine (TPTZ, ≥ 99%), hydrochloric acid (HCl, 37%), sodium acetate trihydrate (C_2_H_3_NaO_2_·3H_2_O), iron(III) chloride hexahydrate (FeCl_3_·6H_2_O), (±)‐6‐hydroxy‐2,5,7,8‐tetramethylchroman‐2‐carboxylic acid (Trolox, ≥ 97%), copper(II) chloride dihydrate (CuCl_2_·2H_2_O), neocuproine (Nc, ≥ 98%), ammonium acetate (C_2_H_7_NO_2_, ≥ 98%), tetrabutylammonium perchlorate (TBAP), dimethylformamide (DMF), dimethyl sulfoxide (DMSO), and ethanol (CAS: 64‐17‐5) were purchased from Sigma‐Aldrich and Merck. Ultrapure water (resistivity ≥ 18.2 MΩ·cm) obtained from a Milli‐Q purification system was used throughout all experiments.

### 2.2. Instrumentation

Elemental analyses were performed with a LECO CHNS‐932 analyzer. The PerkinElmer Spectrum 100 FT‐IR Spectrophotometer was used to record the infrared spectra in the 4000‐400 cm^−1^ spectral region using the attenuated total reflection (ATR) sampling accessory. At ambient temperature, magnetic susceptibility measurements were made using an Alfa magnetic susceptibility balance. The melting points of all the compounds were determined on an EZ‐Melt Automated Melting Point Apparatus. Molar conductances of the complexes in DMF were measured using a direct reading of a WTW 3110 conductivity meter in DMF at 25°C, using concentrations of 1.0 × 10^−3^ M for the complexes. The thermal properties of the complexes were investigated by simultaneous thermogravimetric and differential thermal analyses (TG/DTA) under a nitrogen atmosphere using a Shimadzu TG‐DTA 60 thermal analyzer, employing a heating rate of 10°C min^−1^.

### 2.3. General Method of Synthesis of the Heteroligand Metal Complexes (1–3)

**Scheme 1:**
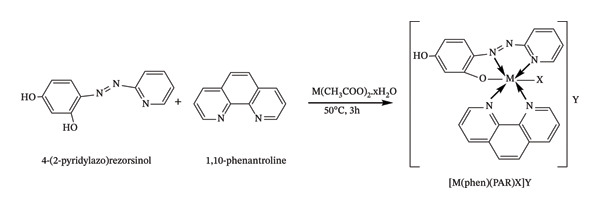
Synthesis of [M(phen) (PAR)X]Y (M = Mn(II), Co(II), Cu(II) and X = AcO in Mn and Cu complexes, H_2_O in Co complex, Y = H_2_O in Mn and Cu complexes, AcO·H_2_O in Co complex).

The studied mixed‐ligand complexes were synthesized using modified methods from the literature [30, 31]. The schematic representation of synthesis is shown in Scheme 1.

Dissolve 20 mL of the azo ligand PAR in acetone (0.430 g, 2 mmol). Subsequently, heat under reflux at 50°C with stirring for approximately 30 min. After complete dissolution of the azo ligand, add the metal salt (2 mmol; Mn(CH_3_COO)_2_·4H_2_O, 0.490 g; Co(CH_3_COO)_2_·4H_2_O, 0.498 g; Cu(CH_3_COO)_2_·H_2_O, 0.399 g) in 15 mL methanol and 2 mmol 1,10‐phenanthroline (0.396 g) in 15 mL acetone. The mixtures were then refluxed at 50°C for 4 h to complete the reaction. After standing for one day, dark‐colored precipitates were obtained. The filtered precipitates were washed with cold water and cold ethanol and dried over P_4_O_10_ before analysis.

### 2.4. Antioxidant Capacity

The ferric‐reducing antioxidant power (FRAP) assay measures antioxidant activity based on the ability of a compound to reduce the Fe^3+^–TPTZ complex to the intensely blue‐colored Fe^2+^–TPTZ form under acidic conditions [32]. The FRAP working solution was freshly prepared by mixing 300 mM acetate buffer (pH 3.6), 10 mM TPTZ solution in 40 mM HCl, and 20 mM FeCl_3_·6H_2_O in a ratio of 10:1:1 (v/v/v). Briefly, 20 μL of 0.1 mM ligand or metal complex solution was mixed with 280 μL of the FRAP reagent. The mixture was vortexed and incubated at 37°C for 30 min to allow complete color development. Absorbance was measured at 593 nm against a reagent blank. Trolox was used as the reference standard.

The cupric ion reducing antioxidant capacity (CUPRAC) assay was performed according to Apak et al. [33]. The method is based on the reduction of Cu(II)‐neocuproine (Nc) complex to Cu(I)‐Nc chelate by antioxidant compounds. The reaction mixture consisted of 250 μL of 10 mM CuCl_2_.2H_2_O, 250 μL of 7.5 mM Nc solution in ethanol, 250 μL of 1.0 M ammonium acetate buffer (pH 7.0), 25 μL of sample solution (in DMSO), and 250 μL of distilled water. Trolox was used as a reference antioxidant. The mixture was incubated at room temperature in the dark for 1 h, and absorbance was measured at 450 nm against a blank. Trolox was used as the reference antioxidant.

All measurements were performed in triplicate and repeated in three independent experiments. Results are expressed as mean ± standard deviation (SD). Statistical analysis was carried out using GraphPad Prism (Version 10.6.1, GraphPad Software, San Diego, CA, USA). Differences between groups were evaluated using one‐way analysis of variance (ANOVA) followed by Tukey’s post hoc test to determine statistical significance. Figures and statistical plots were generated in GraphPad Prism.

### 2.5. Electrochemical Analysis

Electrochemical measurements were carried out using an Autolab PGSTAT 302N potentiostat/galvanostat system (Eco Chemie, Netherlands) controlled by NOVA 2.1.4 software. A conventional three‐electrode electrochemical cell system was employed, consisting of a glassy carbon electrode (GCE, 2.0 mm diameter) as the working electrode, a platinum wire as the counter electrode, and an Ag/AgCl electrode as the reference electrode. All measured potential values were recorded relative to the reference electrode. Before each measurement, the GCE surface was polished with alumina powder with grain sizes of 1.0, 0.3, and 0.05 μm, respectively. Then, the electrode was ultrasonically cleaned in distilled water to remove any residual alumina and subsequently dried at room temperature. Additionally, the electrode was rinsed with DMF before each experiment. Cyclic voltammetry (CV) measurements were conducted according to the method by Oztoprak et al. [34]. The experiments were performed in DMF containing 0.10 M TBAP as the supporting electrolyte. Voltammograms were recorded in 2 mM solutions of the synthesized complexes at a scan rate of 100 mV s^−1^ and in the potential range of −1.0 V to +1.2 V. Before each measurement, the solutions were purged with nitrogen for 10 min to remove dissolved oxygen. All measurements were performed at room temperature.

### 2.6. Computational Details

The geometry optimization calculations of all complexes were performed using Gaussian 09 [35] at the DFT/B3LYP/LANL2DZ level of theory. Electronic properties of all complexes were determined using the TD‐DFT method and 6311G(d,p) basis set. In addition, reactivity descriptors were calculated based on the frontier molecular orbital (FMO) energies obtained from DFT calculations. The ionization potential (*I* = −*E*_HOMO_), electron affinity (A = −*E*_LUMO_), electronegativity (*χ* = (*I* + *A*)/2), chemical potential (*μ* = −(*I* + *A*)/2), and chemical hardness (*η* = (*I* − *A*)/2) values of the complexes were determined.

All complexes were prepared as a ligand using AutoDock Tools 1.5.7. The receptors were selected from protein structures frequently used in antioxidant studies. Kelch‐like ECH‐associated protein 1 (Keap1) (PDB: 5CGJ) and xanthine oxidase (XO) (PDB: 1FIQ) were downloaded from the Protein Data Bank. Receptors were prepared to remove water and ions and add polar hydrogens using AutoDock Tools 1.5.7. After all the preparation steps were completed, the grid boxes were adjusted. The grid box coordinates were determined according to the binding position of the cocrystallized reference ligand in the crystal structure for Keap1, whereas for XO, they were centered on the macromolecule to identify the most favorable binding regions. AutoDock Vina scoring function was used to estimate binding affinities of the complexes. Molecular docking analyses were carried out using the AutoDock Vina program [36].

Molecular dynamics analysis was conducted to compare the stability and interaction patterns of copper (Cu), cobalt (Co), and manganese (Mn) complexes of the same core ligand within identical receptor pockets. Two distinct receptors were chosen for this study, and 100 ns simulations were performed under NPT conditions at 300 K using Schrödinger Desmond software [37–39]. The analysis focused on RMSD and various contact types, including H‐bonding, hydrophobic interactions, ionic bonds, and water‐bridge contacts.

The secondary structure compositions of Keap1 protein are similar across all three complexes, with β‐strand content around 46%–47% and no detectable helices, indicating comparable stability within the globular layer. Ligand charges are −2 for Cu/Mn and −1 for Co. To neutralize the Cu and Mn complex systems, 29 Na^+^ and 22 Cl^−^ ions were added, whereas for the Co complex system, only 6 Na^+^ ions were included. This pattern aligns with the observed increase in ionic and water‐bridge interactions in Cu/Mn complexes and hydrophobic/*π* interactions in the Co complex.

The secondary structure distribution of XO protein, with approximately 21.17% β‐sheets and 27.52% α‐helices, is similar across all three systems. To neutralize the Cu and Mn complex systems, 86 Na^+^ and 94 or 95 Cl^−^ ions were added, whereas only 8 Cl^−^ ions were sufficient for the Co complex system. Ionic and water‐bridge contacts tend to be more continuous in the Cu and Co (net −2 charge) complexes, while hydrophobic and *π* interactions are relatively more prominent in the Mn (net −1 charge) complex.

## 3. Results and Discussion

Bidentate azo ligand (PAR) forms heteroligand metal complexes with Mn(II), Co(II), and Cu(II) in the presence of an N‐donor heterocyclic ligand (phen). The elemental analysis results of the synthesized metal complexes show close agreement with the calculated values, supporting the proposed molecular formulas. Elemental analysis studies revealed that the metal: azo ligand:phen ratio of the complexes was 1:1:1. All complexes are dark‐colored, stable, have high melting points, and are soluble in DMF and DMSO solvents. The physical and analytical details of the prepared complexes are summarized in Table 1.

**TABLE 1:** Physical properties and elemental analysis results of the synthesized complexes.

Compound	MW	Yield (%)	MP (°C)	Color	ΛM (Ω^−1^ cm^2^ mol^−1^)	μeff (B.M.)	Calculated/found			
							C	H	N	M
C_25_H_21_O_5_N_5_Mn (Complex 1) [Mn(phen) (PAR)AcO]·H_2_O	526.38	27	> 350	Dark purple	15.8	5.96	57.04 57.31	4.02 3.88	13.30 13.78	10.44 10.52

C_25_H_23_O_6_N_5_Co (Complex 2) [Co(phen) (PAR)H_2_O]AcO·H_2_O	548.39	36	> 350	Dark brown	68.3	1.68	54.75 54.86	4.22 3.84	12.77 13.13	10.75 10.67

C_25_H_21_O_5_N_5_Cu (Complex 3) [Cu(phen) (PAR)AcO]·H_2_O	534.99	25	> 350	Burgundy	14.9	1.75	56.13 55.81	3.95 3.78	13.09 13.18	11.88 11.64

The molar conductivity values of the complexes, soluble in DMF solvent in 1.0 × 10^−3^ M solution at 25°C, were determined as 68.8 for Co(II), 15.8, 14.9 Ω^−1^ cm^2^ mol^−1^ for Mn(II) and Cu(II), respectively. These significant results showed that the Co(II) complex was an electrolyte in a 1:1 ratio, while the Mn(II) and Cu(II) complexes were not electrolytes [40, 41].

### 3.1. IR Spectra

FT‐IR spectra of the ligand and its metal complexes were recorded in the 4000–400 cm^−1^ region to investigate their coordination behavior (Table 2). The free PAR ligand exhibits a broad absorption band at 3083 cm^−1^, assigned to hydrogen‐bonded phenolic (–OH) groups. The absence of this band in the complexes indicates deprotonation of one phenolic hydroxyl group before coordination [42].

**TABLE 2:** FT‐IR spectroscopic data of the azo ligand (PAR) and the synthesized complexes.

Compound	ʋOH	C=N	N=N	Phen	AcO	C‐O	M–N	M–O
PAR	3083	1628	1592	—		1179	—	—
C_25_H_21_O_5_N_5_Mn (Complex 1) [Mn(phen) (PAR)AcO]·H_2_O	—	1588	1545	1475	1347,1544	1198	565	496
C_25_H_23_O_6_N_5_Co (Complex 2) [Co(Phen) (PAR)H_2_O]AcO·H_2_O	—	1584	1511	1466	1439,1556	1231	592	443
C_25_H_21_O_5_N_5_Cu (Complex 3) [Cu(Phen) (PAR)AcO]·H_2_O	—	1590	1550	1474	1385,1508	1208	598	492

The involvement of the deprotonated phenolic oxygen atom in metal binding is supported by the shift of the *ν* (C–O) stretching vibration from 1179 cm^−1^ in the free ligand to higher frequencies (1198–1231 cm^−1^) in the complexes [43]. Further, the *ν* (N=N) azo stretching band shifts from 1592 cm^−1^ to lower frequencies (1511–1550 cm^−1^), suggesting coordination through the azo nitrogen atom [44–46]. A similar downward shift is observed for the *ν*(C=N) vibration of the pyridylazo moiety, which moves from 1628 cm^−1^ to 1584–1590 cm^−1^ upon complexation. These spectral changes indicate that PAR behaves as a monobasic tridentate ligand, coordinating via phenolic oxygen, azo nitrogen, and pyridyl nitrogen donor atoms [16, 42, 47–49].

The coordination of 1,10‐phenanthroline is evidenced by the shift of the *ν*(C–N) stretching band from 1422 cm^−1^ in the free ligand to 1466–1475 cm^−1^ in the complexes, confirming the involvement of its ring nitrogen atoms in coordination [50–52].

The coordination mode of the acetate group can be assessed from the infrared spectral data by examining the asymmetric and symmetric stretching vibrations of the carboxylate group. The difference between these two vibrations (Δν = ν_as_(COO^−^) − ν_s_(COO^−^)) is commonly used to distinguish between monodentate and bidentate coordination modes. In Complexes 1 and 3, the new bands observed at 1544 and 1347 cm^−1^ for Complex 1 and at 1508 and 1385 cm^−1^ for Complex 3 were assigned to ν_as_ (COO^−^) and ν_s_ (COO^−^) vibrations of the acetate group, respectively. The relatively large separation between these bands supports the monodentate coordination mode of the acetate anion in both complexes, consistent with literature reports [49, 50, 53–57]. In the IR spectrum of Complex 2, bands observed at 1556 and 1439 cm^−1^ were assigned to the asymmetric *ν*_as_ (COO^−^) and symmetric *ν*_*s*_ (COO^−^) stretching vibrations of the acetate ion, respectively. The positions of these bands are characteristic of a noncoordinated (ionic) acetate group. This assignment is further supported by the molar conductivity value of Complex 2, which indicates its electrolytic nature and is consistent with the presence of ionic acetate as a counter ion.

In the low‐frequency region of the spectra (650–400 cm^−1^), new bands attributable to metal–ligand vibrations were observed. The bands at 565, 592, and 598 cm^−1^ for the Mn(II), Co(II), and Cu(II) complexes, respectively, were assigned to *ν* (M–N) stretching vibrations. In addition, the bands observed at 496, 485, and 492 cm^−1^ were attributed to *ν* (M–O) stretching modes for the Mn(II), Co(II), and Cu(II) complexes, respectively. These bands, absent in the spectra of the free ligands, provide further evidence for coordination of the nitrogen and oxygen donor atoms to the metal centers. Overall, the FT‐IR spectral data are consistent with the proposed structures and are in agreement with previously reported studies on related mixed‐ligand metal complexes.

### 3.2. Magnetic Susceptibility Measurements

Magnetic susceptibility measurements, as is well known, provide valuable information about the structure of coordination complexes. The room temperature magnetic moment values indicate that the Mn(II), Co(II), and Cu(II) complexes are paramagnetic in nature. The Mn(II) complex exhibits a magnetic moment of 5.96 B.M., consistent with a high‐spin d^5^ electronic configuration and supporting an octahedral geometry with five unpaired electrons, in agreement with literature values [26, 58]. The Co(II) complex shows a significantly lower magnetic moment of 1.68 B.M., which deviates from the typical high‐spin octahedral Co(II) values (ca. 4.3–5.2 B.M.). Such a reduction may be attributed to strong ligand field effects, possible orbital angular momentum quenching, or solid‐state magnetic interactions, suggesting a distorted coordination environment rather than an idealized geometry. The Cu(II) complex displays a magnetic moment of 1.75 B.M., consistent with a d^9^ system (*S* = 1/2), which is commonly observed for octahedral or Jahn–Teller distorted octahedral geometries. Overall, the magnetic data, together with spectroscopic (FT‐IR), thermal (TGA), conductivity, and theoretical (DFT) results, collectively support the proposed coordination environments of the complexes [59–61].

### 3.3. Thermal Analyses

**FIGURE 1:**
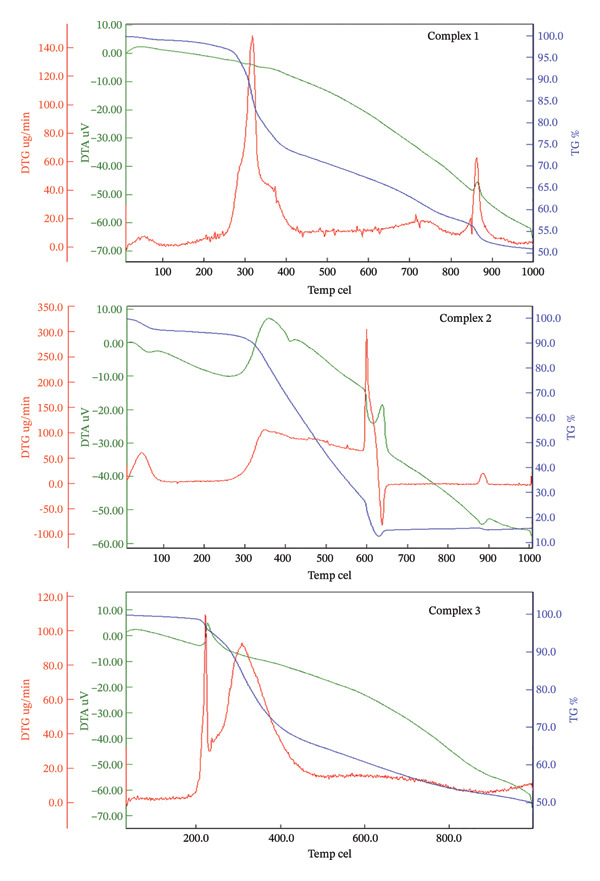
DTA/TG thermograms of the synthesized complexes.

The thermal behavior of Complexes 1–3 was investigated by TG/DTG analysis between 25°C and 1000°C under a nitrogen atmosphere at a heating rate of 10°C min^−1^. The TG/DTG curves are shown in Figure 1, and the thermal data are summarized in Table 3, providing insight into the thermal stability and decomposition behavior of the complexes.

**TABLE 3:** Thermal analysis (DTA/TG) results of the synthesized complexes.

Complex	TG range (°C)	DTA_max_ (°C)	Calculated (estimated) mass loss (%)		Assignment	Metallic residue
			Mass loss	Total mass loss		
1	25–235		3.41 (3.20)		Loss of a lattice water	
	235–890	355, 865	45.45 (44.25)	48.86 (47.45)	Loss of acetate and phen	
	890–1000				Loss of the other groups	Mn‐containing residue

2	25–65		3.28 (3.72)		Loss of an lattice water	
	65–355		14.05 (14.38)		Loss of acetate and a coord. water	
	355–1000	385, 635	69.00 (66.12)	86.33 (84.22)	Loss of the other groups	CoO 13.67 (15.78)

3	25–200		3.37 (3.12)		Loss of a lattice water	
	200–870	225	44.71 (44.00)	48.08 (47.12)	Loss of acetate and phen	
	870–1000				Loss of the other groups	Cu‐containing residue

For Complex 1, [Mn(PAR) (Phen)AcO]·H_2_O, the first mass loss of 3.20% (calcd. 3.41%) between 25°C and 235°C corresponds to the release of lattice water. The second stage (235°C–890°C) with a mass loss of 44.25% (calcd. 45.45%) is attributed to the removal of acetate (AcO^−^) and phen ligands. The decomposition was not completed up to 1000°C, indicating the formation of a thermally stable residue.

For Complex 2, [Co(PAR) (Phen) (H_2_O)]AcO·H_2_O, the first step (25°C–65°C) shows a mass loss of 3.72% (calcd. 3.28%) due to lattice water removal. The second stage (65°C–355°C) with a mass loss of 14.38% (calcd. 14.05%) is assigned to the loss of acetate and coordinated water. The final residue was identified as cobalt(II) oxide (CoO), with an experimental yield of 15.78% in reasonable agreement with the calculated value of 13.67%.

For Complex 3, [Cu(PAR) (Phen)AcO]·H_2_O, the first decomposition step (25°C–200°C) shows a mass loss of 3.12% (calcd. 3.37%) corresponding to lattice water removal. The second stage (200°C–870°C) with a mass loss of 44.00% (calcd. 44.71%) is attributed to the elimination of acetate and phen ligands. The decomposition was not completed up to 1000°C, suggesting the formation of a stable residue under nitrogen atmosphere.

### 3.4. Antioxidant Activity

Antioxidant activity refers to the ability of a molecule to neutralize or inhibit free radicals, thereby preventing oxidative damage to biomolecules and maintaining redox homeostasis [62]. In this context, evaluating the antioxidant potential of metal complexes is particularly important, as their intrinsic redox‐active metal centers enable efficient electron‐transfer processes that mitigate oxidative stress and contribute to cytoprotection [63, 64]. Moreover, the metal–ligand coordination environment plays a critical role in modulating redox potential and radical‐scavenging efficiency. A detailed understanding of these interactions is essential for the rational design of new metal‐based compounds with enhanced bioactivity and therapeutic potential [65]. To assess the antioxidant potential of such systems, several analytical methods are commonly employed. Among these, the FRAP and CUPRAC assays are widely preferred for evaluating both ligands and their metal complexes due to their reliability, simplicity, and broad applicability. These complementary methods provide valuable insights into the electron‐transfer capacity of the studied compounds under different redox conditions.

Although both FRAP and CUPRAC assays are based on electron‐transfer mechanisms, they differ significantly in their operating conditions and redox chemistry. The FRAP assay is conducted under acidic conditions (pH 3.6) and measures the ability of compounds to reduce the Fe^3+^–TPTZ complex to Fe^2+^–TPTZ, thereby reflecting FRAP. In contrast, the CUPRAC method operates at near‐neutral pH and is based on the reduction of the Cu^2+^–neocuproine complex to Cu^+^–neocuproine, providing a more physiologically relevant evaluation of antioxidant capacity. Due to these differences, the two assays may yield variations in the relative antioxidant performance of the tested compounds. Such discrepancies arise from differences in metal–ligand interactions, redox potentials, and the electron‐donating ability of the complexes under distinct conditions. Therefore, the combined use of FRAP and CUPRAC assays enables a more comprehensive understanding of the redox behavior and antioxidant mechanisms of the studied metal complexes.

**FIGURE 2:**
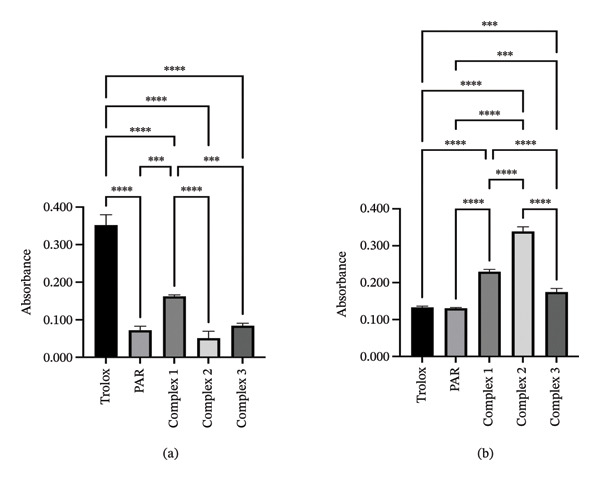
FRAP (a) and CUPRAC (b) assays comparing the antioxidant capacities of Trolox, PAR, and the metal complexes, presented as mean ± SD (*n* = 3). Statistical differences between groups are indicated by significance markers (^∗∗∗^ *p* < 0.001, ^∗∗∗∗^ *p* < 0.0001).

The antioxidant capacities of the ligands and their corresponding metal complexes were evaluated using the FRAP assay, with Trolox as the reference standard, and EC_50_ values, and the results are summarized in Table 4. The EC_50_ value was defined as the concentration of the complex required to achieve an absorbance of 0.5 [66]. In this method, an increase in absorbance at 593 nm is directly associated with enhanced ferric‐reducing antioxidant activity. All samples were analyzed at the same concentration (0.1 mM) to ensure a reliable comparison. As expected for the positive control, Trolox exhibited the highest reducing capacity among all tested samples (Figure 2a).

**TABLE 4:** Comparison of the reducing activities of 0.1 mM samples determined by the FRAP and CUPRAC assays.

Sample	FRAP		CUPRAC			
	λ_593_ ^∗^	*R* ^2^	EC_50_ (mM)	λ_450_ ^∗^	*R* ^2^	EC_50_ (mM)
PAR	0.072 ± 0.007	0.9967	0.100	0.131 ± 0.002	1	0.038
Phen	—	—	—	—	—	—
Complex 1	0.162 ± 0.004	0.9963	0.051	0.232 ± 0.005	0.9999	0.021
Complex 2	0.051 ± 0.016	0.9994	0.084	0.339 ± 0.009	0.9996	0.017
Complex 3	0.082 ± 0.006	0.9932	0.092	0.172 ± 0.007	0.9999	0.029
Trolox	0.352 ± 0.028	0.9996	0.014	0.132 ± 0.004	0.9998	0.039

^∗^Absorbance values.

Among the tested samples, Complex 1 demonstrated the highest ferric‐reducing activity (absorbance: 0.162), showing significantly greater activity than PAR and Complex 3, as well as a statistically meaningful difference compared with Complex 2. In contrast, PAR and Complex 3 displayed comparable activities with no statistically significant difference (ns), indicating similar reducing capacities, whereas Complex 2 exhibited the lowest FRAP response, confirming its relatively weak antioxidant potential. At this concentration, the phen ligand showed no detectable activity. Overall, PAR demonstrated a lower reducing capacity than its metal complexes—except for Complex 2—suggesting that metal coordination enhances the redox‐based antioxidant performance of the ligand. Although Complex 1 emerged as the most active compound among the tested ligands and complexes, all samples exhibited lower antioxidant capacity than Trolox under the experimental conditions.

In the CUPRAC assay, the antioxidant capacity was evaluated based on absorbance at 450 nm. Consistent with the FRAP results, the phen ligand showed no detectable antioxidant activity, whereas the PAR ligand exhibited a cupric‐reducing capacity comparable to that of Trolox (Figure 2b). In contrast, all metal complexes demonstrated significantly enhanced CUPRAC activity relative to the free ligand, indicating that coordination to the metal center improves electron‐transfer efficiency. Among the synthesized complexes, Complex 2 exhibited the highest antioxidant activity, with absorbance values consistently exceeding 0.339, highlighting its superior cupric‐reducing potential under the tested conditions. Complex 1 showed the second‐highest activity, followed by Complex 3, which displayed moderate reducing capacity. Overall, PAR presented the lowest activity among the complexes and ligands, remaining significantly weaker than its corresponding metal complexes. These findings demonstrate that metal coordination substantially enhances redox‐based antioxidant performance, with Complex 2 emerging as the most potent antioxidant, and underscore the critical role of metal–ligand interactions in modulating electron‐transfer processes.

Although both FRAP and CUPRAC assays are widely used to assess antioxidant activity, they rely on distinct different redox mechanisms, which may lead to differences in the relative ranking of the samples. The FRAP assay measures the ability of compounds to reduce Fe^3+^ to Fe^2+^ under acidic conditions, whereas the CUPRAC method evaluates the reduction of Cu^2+^ to Cu ^+^ at near‐physiological pH. These differences influence how each complex interacts with the redox system, depending on its coordination environment and intrinsic electron‐transfer properties. Accordingly, Complex 1 exhibited the highest activity in the FRAP assay, while Complex 2 demonstrated superior performance in the CUPRAC system.

The antioxidant behavior observed for the synthesized PAR–phen metal complexes is consistent with previous studies demonstrating that metal coordination can significantly enhance the redox properties of organic ligands. Several reports have shown that transition metal complexes containing 1,10‐phenanthroline exhibit stronger antioxidant activity than their corresponding free ligands owing to improved electron‐transfer capability and stabilization of radical intermediates. For example, Neykov et al. reported that Pd(II)‐phenanthroline complexes displayed significantly higher antioxidant activities than free phenanthroline derivatives in both HORAC and ORAC assays [67]. Similarly, Ashrafuzzaman et al. demonstrated that mixed‐ligand Cu(II), Ni(II), and Zn(II) complexes containing 1,10‐phenanthroline exhibited enhanced DPPH radical‐scavenging activities compared with the corresponding free Schiff base ligand [68]. Among the investigated complexes, the Cu(II) derivative showed the highest antioxidant activity (IC_50_ = 61.88 μg/mL), whereas the Ni(II) and Zn(II) complexes displayed lower activities (IC_50_ = 139.66 and 510.20 μg/mL, respectively). The superior activity of the Cu(II) complex was attributed to its favorable redox properties and enhanced electron‐transfer capability. Consistent with these findings, coordination of PAR with Mn(II), Co(II), and Cu(II) ions in the present study resulted in a marked enhancement of antioxidant activity compared with the free ligand. Complex 1 exhibited the highest ferric‐reducing capacity in the FRAP assay (0.162 ± 0.004), corresponding to an approximately 2.3‐fold increase relative to PAR (0.072 ± 0.007), whereas Complex 2 displayed the highest CUPRAC activity (0.339 ± 0.009), representing an approximately 2.6‐fold enhancement compared with PAR (0.131 ± 0.002). Similar improvements have also been reported for metal–flavonoid systems, where complexes of quercetin, rutin, and morin showed superior antioxidant activities compared with the corresponding free flavonoids due to enhanced electron‐donating ability and stabilization of phenoxyl radical intermediates [69–71]. Therefore, the enhanced antioxidant activity observed for the PAR–phen complexes can be attributed to coordination‐induced modifications in the electronic structure of the ligand, which facilitate electron‐transfer processes and stabilize reactive radical species. The different activity rankings observed in the FRAP and CUPRAC assays may further reflect differences in metal‐centered redox potentials, coordination environments, and electron‐transfer pathways under the respective assay conditions, as previously reported for transition metal complexes [63, 72, 73]. Overall, the obtained results are in good agreement with previously reported metal–organic antioxidant systems and confirm that metal coordination plays a crucial role in enhancing the antioxidant properties of PAR‐based ligands.

### 3.5. Electrochemical Properties

**FIGURE 3:**
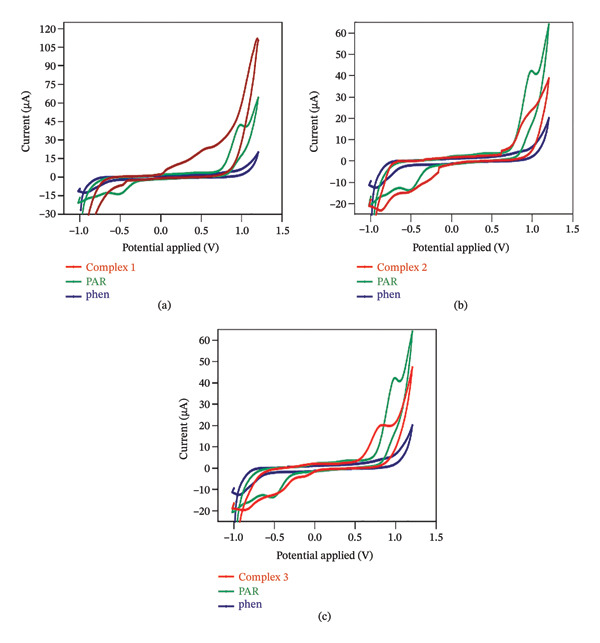
Cyclic voltammograms of PAR, phen, and Complexes 1–3 in TBAP/DMF.

The electrochemical behavior of the synthesized heterometallic Complexes (1–3) with PAR and 1,10‐phenanthroline (phen) ligands was investigated by CV in DMF. Measurements were carried out in solutions containing 0.1 M TBAP as the supporting electrolyte within a potential range of −1.0 and + 1.2 V. All voltammograms were recorded at a scan rate of 100 mV s^−1^, and the cyclic voltammograms of the free ligands and their corresponding metal complexes are presented in Figure 3a–c. The obtained results reveal electrochemical differences between the ligands and the metal complexes. The absence of any pronounced oxidation or reduction peak in the voltammogram of the phen ligand in the investigated potential range indicates that this ligand is electrochemically inactive under the applied experimental conditions. In contrast, the PAR ligand exhibited a well‐defined oxidation peak at 0.983 V, confirming its electroactive nature. The observation of oxidation potentials for the metal complexes at values different from those of the free ligands indicates that the metal ions were successfully coordinated to the ligand environment and that complex formation is supported by the electrochemical data. The oxidation of Complex 1 at 0.550 V, which is significantly lower than that of the PAR ligand, suggests that Mn coordination markedly facilitates electron transfer (Figure 3a). This pronounced cathodic shift indicates that metal–ligand interactions lead to a redistribution of electron density within the complex structure and facilitate the oxidation process. Complex 2 exhibited an oxidation peak at 0.953 V, giving an electrochemical response at a potential quite close to that of the PAR ligand (Figure 3b). However, this partial shift in oxidation potential still indicates that incorporation of the Co ion into the coordination sphere results in a change in the electronic structure of the complex. The oxidation of Complex 3 at 0.822 V further shows that Cu coordination also facilitates the oxidation process (Figure 3c). The fact that the oxidation potential of Complex 3 is lower than that of Complex 2 but higher than that of Complex 1 demonstrates that the nature of the metal center and the metal–ligand binding characteristics play a decisive role in the electrochemical behavior. In conclusion, the oxidation processes observed at different potentials compared with those of the free ligands strongly support the formation of the synthesized metal complexes and reveal that Mn, Cu, and Co ions influence the redox behavior to different degrees after coordination. These findings are important in showing that the complexes differ markedly from the free ligands not only structurally but also in terms of their electrochemical properties.

**FIGURE 4:**
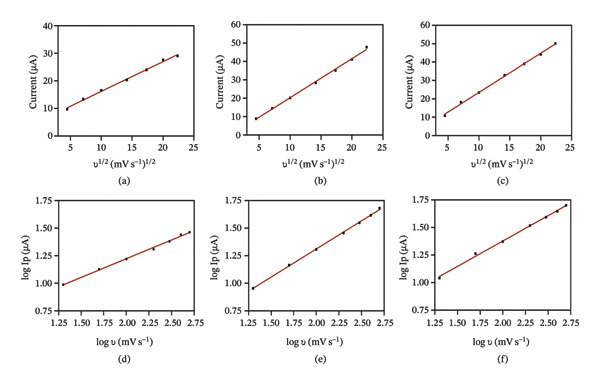
Ipa–*ν* ^1/2^ and log Ipa–log v plots for Complex 1 (a, d), Complex 2 (b, e), and Complex 3 (c, f).

CV measurements were performed at varying scan rates (20–500 mV s^−1^) to evaluate the effect of the potential scan rate (*ν*) on the electrochemical response of the complexes [31]. According to the linear regression data presented in Table 5, a linear relationship was observed between the anodic peak current (Ipa) and the square root of the scan rate (*ν* ^1/2^) for all complexes, and the correlation coefficients were determined to be approximately *R* ^2^≈0.99 (Figure 4a–c). This relationship indicates that the electrochemical process is diffusion‐controlled. Furthermore, the slopes of the log Ipa versus log ʋ plots were found to be close to 0.5, further confirming that electrochemical processes are predominantly governed by diffusion (Figure 4d–f). These findings demonstrate that the redox behavior is controlled by the diffusion of electroactive species from the bulk solution to the electrode surface, rather than by surface‐confined processes.

**TABLE 5:** Regression equations for Ip vs. *ʋ* ^1/2^ and log Ip vs. log ʋ plots of Complexes (1–3) cyclic voltammetry data.

	Ip = *ʋ* ^1/2^	log Ip = log ʋ
Complex 1	Ipa (μA) = 1.0782 *ʋ* ^1/2^ + 5.3794 (*R* ^2^ = 0.9958)	log Ipa (μA) = 0.3398 log ʋ + 0.5427 (*R* ^2^ = 0.9970)
Complex 2	Ipa (μA) = 2.1227 *ʋ* ^1/2^ − 0.8079 (*R* ^2^ = 0.9977)	log Ipa (μA) = 0.5102 log ʋ − 0.2901 (*R* ^2^ = 0.9991)
Complex 3	Ipa(μA) = 2.1315 *ʋ* ^1/2^ + 2.2881 (*R* ^2^ = 0,9982)	log Ipa(μA) = 0.4597 log ʋ + 0.4571 (*R* ^2^ = 0,9974)

### 3.6. Geometry Optimization

**FIGURE 5:**
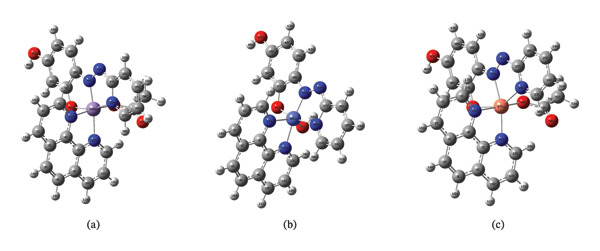
The optimized geometries of Complex 1 (a), Complex 2 (b), and Complex 3 (c).

The optimized molecular structures of the all complexes are shown in Figure 5a–c. The calculated energies of Complexes 1, 2, and 3 were −1642.586,242 a.u., −1531.673,560 a.u., and −1734.742,496 a.u., respectively. Optimized parameters for all three complexes are given in Table 6.

**TABLE 6:** The obtained optimization parameters of all complexes with the DFT method B3LYP/LANL2DZ theory level.

	Complex 1		Complex 2		Complex 3	
Bond length	N21‐Mn54	2.04546	N21‐Co48	1.99629	N21‐Cu47	2.12160
	N22‐Mn54	2.05509	N22‐Co48	2.00876	N22‐Cu47	2.07869
	N28‐Mn54	2.00978	N28‐Co48	1.92177	N28‐Cu47	2.30686
	N34‐Mn54	1.87816	N34‐Co48	1.81037	N34‐Cu47	2.09812
	O44‐Mn54	1.96897	O48‐Co48	1.95485	O48‐Cu47	1.96722
	N22‐C13	1.34842	N22‐C13	1.34531	N22‐C13	1.34330
	N22‐C7	1.37642	N22‐C7	1.37379	N22‐C7	1.37087
	N21‐C5	1.34584	N21‐C5	1.34252	N21‐C5	1.34044
	N21‐C4	1.37163	N21‐C4	1.37047	N21‐C4	1.36841

Bond angle	N21‐Mn54‐N22	80.22481	N21‐Co48‐N22	82.54117	N21‐Cu47‐N22	79.81534
	N34‐Mn54‐N28	79.29961	N34‐Co48‐N28	81.19226	N34‐Cu47‐N28	73.79791
	N34‐Mn54‐O44	83.88836	N34‐Co48‐O44	84.80823	N34‐Cu47‐O44	87.48959

When the bonds between the N atoms of 1,10‐phenanthroline and the Mn atom in the complex were examined, the Mn–N bond lengths were determined to be 2.04546 and 2.05509 Å, respectively, and the N21–Mn54–N22 bond angle was measured as 80.22481°. The N atoms of the 1,10‐phenanthroline moiety in the complex have bond lengths of 1.34584 Å (C5‐ 21N) and 1.34842 Å (C13‐ 22N) with the C atoms. In a study where a Mn complex was formed with 1,10‐phenanthroline, the bond length between Mn and N was calculated as 2.025 Å and the N‐Mn‐N angle as 75.55° in the structure optimized by the DFT method [74]. In another study, N‐Mn bond lengths were reported to be in the range of 2.04–2.06 Å [75]. Although the theoretical levels and complexes are different, it was determined that the study using the DFT method and this study yielded similar values.

When the bonds between the N atoms of 1,10‐phenanthroline and the Co atom in the complex were examined, the Co–N bond lengths were determined to be 1.99629 and 2.00876 Å, respectively, and the N21–Co48–N22 bond angle was measured as 82.54117°. When we look at the part where the Co complex is bonded with PAR, the value of the N34‐Co48‐N28 angle was determined as 81.19226°, and the bond lengths were calculated as 1.81037 and 1.92177 Å for N34‐Co48 and N28‐Co48, respectively.

When the bonds between the N atoms of 1,10‐phenanthroline and the Cu atom in the complex were examined, the Cu–N bond lengths were determined to be 2.07869 and 2.1216 Å, which are consistent with values found in the literature for similar complex structures (1.9984; 2.1205 Å) [76]. The N21–Cu–N22 bond angle was measured as 79.81534 °, which is very close to the literature (81.40°). The N atoms of the 1,10‐phenanthroline moiety in the complex have bond lengths of 1.34 and 1.37 Å with the C atoms. These values are in full agreement with theoretical values in the literature [76] and are also highly consistent with experimental XRD results [77]. Among the bonds between PAR and Cu in the complex, the bond lengths between N atoms and Cu are prominent, and the theoretical values were determined as 2.0912 and 2.30686 Å. It was evaluated that the experimental and theoretical values in the literature were compatible with the values obtained in this study [16].

### 3.7. Highest Occupied Molecular Orbital–Lowest Unoccupied Molecular Orbital (HOMO‐LUMO) Analysis

**FIGURE 6:**
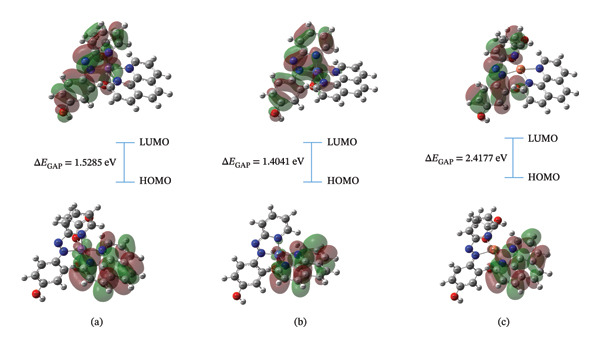
Frontier molecular orbitals (HOMO, LUMO) of the Complex 1 (a), Complex 2 (b), and Complex 3 (c).

The HOMO‐LUMO energy values calculated after optimization provide important insights into the chemical reactivity and kinetic stability of the molecules [78]. The FMO analysis was performed using the TD‐DFT method, and the corresponding HOMO and LUMO distributions of the complexes are presented in Figure 6a–c. According to the calculated results, the HOMO energy level is an indicator of the electron donor capacity and reveals the location of the electrons concentrated in the molecule’s HOMO. Conversely, the LUMO energy level represents the molecule’s electron acceptance potential.

The HOMO energies for Complexes 1, 2, and 3 were calculated as −4.1957 eV, −4.05885 eV, and −5.0017 eV, respectively, while the LUMO energies were calculated as −2.66726 eV, −2.65474 eV, and −2.58399 eV. ΔE = ELUMO – EHOMO, the energy difference between the HOMO and LUMO levels, is a critical parameter for the molecule’s chemical stability and reactivity. The results of the study show that ΔE values are 1.5285 eV, 1.4041 eV, and 2.4177 eV for Complexes 1, 2, and 3, respectively. The relatively smaller gaps of Complexes 1 and 2 suggest that these molecules are more chemically reactive and more susceptible to electron transfer. In contrast, larger ΔE values are typically associated with greater kinetic stability and lower reactivity. Analysis of the orbital density maps shows that the HOMO is mainly localized over the phen region, whereas the LUMO is distributed on the PAR region. This spatial separation provides important information about the likely nucleophilic and electrophilic attack sites within the molecules.

In addition to the HOMO‐LUMO energy gap, global reactivity descriptors, such as chemical hardness (*η*), chemical potential (*μ*), electronegativity (*χ*), ionization potential (*I*), and electron affinity (*A*), were evaluated to obtain a more quantitative description of molecular reactivity. These parameters provide a deeper insight into the intrinsic chemical reactivity and potential biological activity of the studied complexes. Among the studied complexes, Complex 2 exhibits the lowest chemical hardness, while Complex 3 shows the highest hardness, consistent with its lower reactivity and greater stability (see Table 7).

**TABLE 7:** The calculated values of ionization potential, electron affinity, electronegativity, chemical hardness, and chemical potential of the complexes.

	Complex 1 (eV)	Complex 2 (eV)	Complex 3 (eV)
Ionization potential (*I*)	41,957	405,885	50,017
Electron affinity (*A*)	266,726	265,474	258,399
Electronegativity (*χ*)	343,148	3,356,795	3,792,845
Chemical hardness (*η*)	076,422	0,702,055	1,208,855
Chemical potential (*μ*)	−3,43,148	−3,356,795	−3,792,845

In general, FMO analyses clearly reveal the reactive aspects of the studied molecule’s electronic structure, the charge distribution in the bond structures, and potential interaction sites. These data provide critical information regarding both the molecule’s interaction with biological targets and its chemical stability, supporting the docking and MD results.

### 3.8. Molecular Electrostatic Potential (MEP) Analysis

MEP analysis is considered an important analysis for determining the electrostatic charge distributions of molecules, the positive and negative charge densities in their three‐dimensional structures, and the electrophilic and nucleophilic regions. The resulting MEP surface of the molecules provides a three‐dimensional representation of the molecule’s electrostatic structure, and a spatial representation of the molecular electrostatic environment, where red regions indicate negative potential, blue regions indicate positive potential, and green regions represent nearly neutral areas.

MEP analyses were performed using the Gaussian 09 program for the all complexes.

**FIGURE 7:**
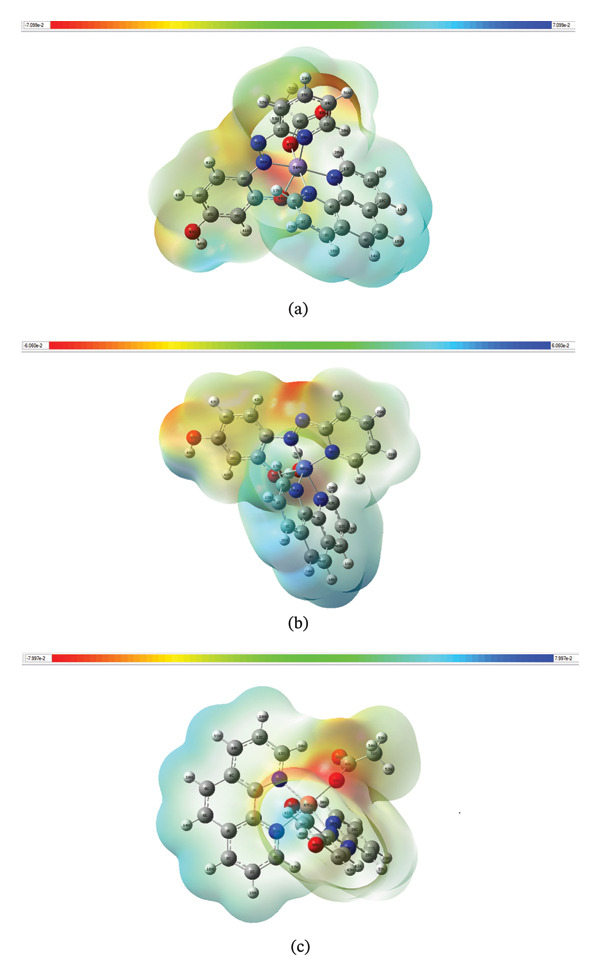
MEP map of Complex 1 (a), Complex 2 (b), and Complex 3 (c).

The results showed that the minimum and maximum potential values were approximately ±0.07099 a.u., ±0.06060 a.u., and ±0.07997 a.u. for Complexes 1, 2, and 3, respectively. Examination of the MEP maps indicated that the most negative regions in Complexes 1 and 3 are localized around the atoms involved in Cu/Mn–acetyl bonding and around atom 44O, where the PAR group coordinates to Cu (Figure 7a, c). Molecular docking studies have also shown that these regions are particularly prone to interaction. Conversely, the region with the most pronounced positive potential is the region where the H46 atom is located. Furthermore, the phen region forming the complex is closer to the positive potential. These positive regions of the molecule played a role in pi interactions and van der Waals interactions in the molecular docking study. For Complex 2, the regions with the most negative potential were observed in the regions where the oxygen and nitrogen in the PAR group of the complex are located. The negative potential of the O45 atom is a crucial point for electrostatic interactions (see Figure 7b). Similarly, the H46 atom, bonded to O45, is located in regions with positive potential and is susceptible to interaction. Molecular docking studies have also shown that the OH group in the PAR group of the complex plays a role in hydrogen bonding interactions.

Overall, MEP analysis provided a clear definition of the electrophile/nucleophile distribution and potential binding sites of the studied molecule. The combined evaluation of the HOMO‐LUMO energy gap and the MEP surface clearly demonstrates how the electronic structure governs the reactivity and preferred interaction sites of the complexes. These results form a critical basis for the docking and MD simulations performed and contribute significantly to understanding the mechanisms of the molecule’s interaction with biological targets.

### 3.9. Molecular Docking

In this study, potential binding interactions between the metal complexes and target proteins were evaluated using molecular docking studies. The molecular docking study aimed to determine the binding affinity and binding pose predictions of the metal complexes identified as ligands for target receptors. The binding affinity values obtained from the calculations revealed the selective binding conformations of the ligands toward the target proteins and provided preliminary information regarding their potential inhibitory potential. The poses with the lowest binding energy were considered the most suitable binding positions.

In the molecular docking study performed with XO, initial docking calculations were primarily carried out by considering the cocrystallized ligand binding site as the reference active site. However, docking within this site resulted in relatively weaker binding affinity values and unfavorable RMSD values for the metal complexes investigated. Therefore, a blind docking analysis encompassing the entire macromolecular structure was subsequently performed, revealing a more favorable binding site near the central cavity of the enzyme, where significantly improved binding affinity and RMSD values were obtained.

**FIGURE 8:**
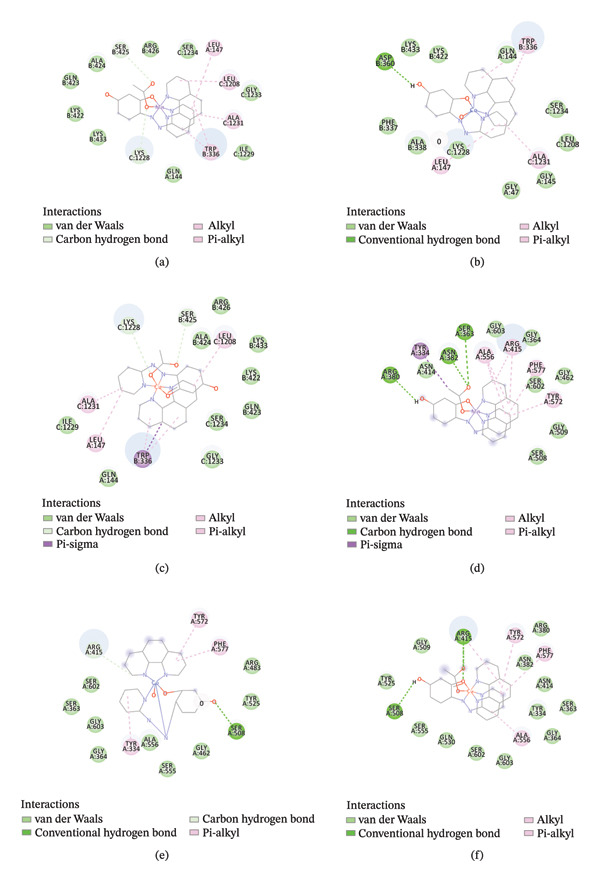
Interaction profiles of Complex 1 (a, d), Complex 2 (b, e), and Complex 3 (c, f) with XO and Keap1.

In the molecular docking study performed with XO, the binding affinity values of the Complexes 1, 2, and 3 were calculated as −9.1 kcal/mol, −10.2 kcal/mol, and −8.5 kcal/mol, respectively (see Figure 8a–c and Table 8). Hydrogen bonding interactions were observed in the binding profile of Complex 2, which exhibited the highest binding affinity value, and this interaction was determined to occur with Asp‐360 residue. Trp‐336 was involved in pi‐alkyl interactions in all three metal complexes. In the Complex 3, Trp‐336 also played a role in pi‐sigma interactions. Ala‐1231 was also identified as a key residue involved in alkyl interactions. Residues Gln‐144, Ser‐1234, and Lys‐422 were identified as common residues in the interaction profiles of Complexes 1, 2, and 3, participating in van der Waals interactions. In the literature, these residues have been implicated in different interactions, such as vdw and H‐bonding interactions [79]. Compared to the study conducted with catechin and gallic acid, Complexes 1, 2, and 3 were found to have better binding affinities [39]. It was also determined that the three complexes interacted with the residues to which gallic acid forms H‐bonds, albeit with different interaction types [80].

**TABLE 8:** Molecular interactions of all complexes with Keap1 receptor and xanthine oxidase receptor as a result of molecular docking.

	Xanthine oxidase receptor			Keap‐1 receptor		
	Complex 3 [Cu(PAR) (Phen)AcO]·H_2_O	Complex 2 [Co(PAR) (Phen) (H_2_O)]AcO·H_2_O	Complex 1 [Mn(PAR) (Phen)AcO]·H_2_O	Complex 3 [Cu(PAR) (Phen)AcO]·H_2_O	Complex 2 [Co(PAR) (Phen) (H_2_O)]AcO·H_2_O	Complex 1 [Mn(PAR) (Phen)AcO]·H_2_O
Binding affinity (kcal/mol)	−8.5	−10.2	−9.1	−8.3	−8.5	−8.9

Protein–ligand contacts (H‐bonds)	—	Asp‐360 (2.22 Å)	—	Arg‐508 (2.57 Å) Arg‐415 (2.79 Å and 2.95 Å)	Ser‐508 (2.83 Å)	Arg‐380 (2.76 Å) Asn‐382 (2.95 Å) Ser‐363 (3.01 Å)

Protein–ligand contacts (pi‐alkyl)	Trp‐336 (4.44 Å and 3.91 Å)	Trp‐336 (4.00 Å, 4.83 Å, 4.53 Å, 4.06 Å)	Trp‐336 (3.85 Å, 4.05 Å, 5.47 Å, 4.41 Å)	Tyr‐572 (5.46 Å) Phe‐577 (5.18 Å)	Tyr‐334 (5.02 Å) Tyr‐572 (4.96 Å) Phe‐577 (5.15 Å)	Phe‐577 (4.90 Å) Tyr‐572 (4.70 Å)

Protein–ligand contacts (pi‐sigma)	Trp‐336 (3.72 Å)	—	—	—	—	Tyr‐334 (3.61 Å)

Protein–ligand contacts (alkyl)	Ala‐1231 (3.34 Å) Leu‐147 (4.89 Å) Leu‐1208 (4.28 Å)	Leu‐147 (4.98 Å) Ala‐1231 (3.82 Å)	Ala‐1231 (3.04 Å) Lys‐1208 (4.36 Å) Leu‐147 (5.41 Å)	Arg‐415 (5.28 Å) Ala‐556 (4.41 Å)	—	Ala‐556 (4.38 Å, 3.63 Å, 4.37 Å) Ala‐415 (4.34 Å and 4.46 Å)

Ligand–protein contacts (carbon H‐bond)	Lys‐1228 (3.26 Å) Ser‐425 (3.42 Å)	—	Leu‐1228 (3.15 Å) Ser‐425 (3.30 Å)	—	Arg‐415 (3.06 Å)	—

Ligand–protein contacts (VdW)	Ile‐1229, Gln‐144, Gly‐1233, Ser‐1234, Gln‐423, Lys‐422, Lys‐433, Ala‐424, Arg‐426	Lys‐433, Lys‐422, Gln‐144, Ser‐1234, Leu‐1208, Gly‐145, Gly‐47, Lys‐1228, Ala‐338, Phe‐337	Ser‐1234, Arg‐426, Ala‐424, Gln‐423, Lys‐422, Lys‐433, Gln‐144, Ile‐1229, Gly‐1233	Gly‐509, Tyr‐525, Ser‐555, Gln‐530, Ser‐602, Gly‐603, Gly‐364, Tyr‐334, Ser‐363, Asn‐414, Asn‐382, Arg‐380	Ser‐602, Ser‐363, Gly‐603, Gly‐364, Ala‐556, Ser‐555, Gly‐462, Tyr‐525, Arg‐483	Asn‐414, Gly‐603, Gly‐364, Ser‐602, Gly‐462, Gly‐509, Ser‐508

The observation that the complexes preferentially bind to an alternative region rather than the cocrystallized ligand site suggests the presence of secondary or alternative binding pockets within XO, a phenomenon that has also been reported for structurally diverse inhibitors in previous computational studies. These findings indicate that the binding profile of the metal complexes at the XO binding site is consistent with literature studies, and Complex 2, in particular, exhibits a stronger binding affinity, making it a prominent potential inhibitor candidate.

For validation of the docking protocol, the cocrystallized ligand of Keap1 (PDB: 5CGJ) was re‐docked into the active site using the same docking parameters and showed a binding affinity value of −11.0 kcal/mol.

Molecular docking studies performed with Keap1 revealed binding affinity values of −8.9, −8.5, and −8.3 kcal/mol for Complexes 1, 2, and 3, respectively. Complex 3 formed hydrogen bonds with residues Arg‐415 and Ser‐508, Complex 2 with residue Ser‐508, and Complex 1 with residues Arg‐380, Asn‐382, and Ser363 (see Figure 8d–f and Table 8). Tyr‐572 and Phe‐577 residues played a role in pi‐alkyl interactions in all three metal complexes. Common residues in van der Waals interactions of all three metal complexes were identified as Gly‐364, Gly‐603, and Ser‐602. Comparison with the interaction profile of the reference ligand in the 5CGJ pdb file indicated that Tyr‐572 and Phe‐577, which also participate in the same interactions in the reference ligand, play important roles in pi‐alkyl interactions in the active site of Keap1 [39]. Other important residues reported in the literature are Tyr‐334, Arg‐380, Arg‐415, Arg‐483, and Tyr‐525 [81]. These residues, along with Complexes 1, 2, and 3, were determined to form bonds, pi interactions, or van der Waals interactions. Although the binding affinity values of the synthesized complexes were lower than that of the cocrystallized reference ligand, the observed interactions with critical active‐site residues indicated a similar binding pattern within the Keap1 binding pocket. These findings demonstrated that the metal complexes had a binding profile consistent with the critical interaction residues in the Keap1 binding region, and Complex 1, in particular, exhibited a stronger binding affinity, suggesting that it may serve as a promising candidate for further investigation as a potential Keap1 inhibitor.

### 3.10. MD Results of Keap‐1 Interactions

Transition metal complexes demonstrate various binding modes within protein pockets, influenced by their charge, coordination, and π‐electronic features. For instance, all complexes sharing the same core ligand can form differing degrees of ionic, H‐bonding, water‐bridging, and hydrophobic or *π* interactions with cationic or acidic sites on the receptor. This work evaluates the standard Simulation Interactions Diagram derived from three 100 ns MD simulations of the same Keap‐1 receptor, analyzing RMSD, contact types, and timescales.

**FIGURE 9:**
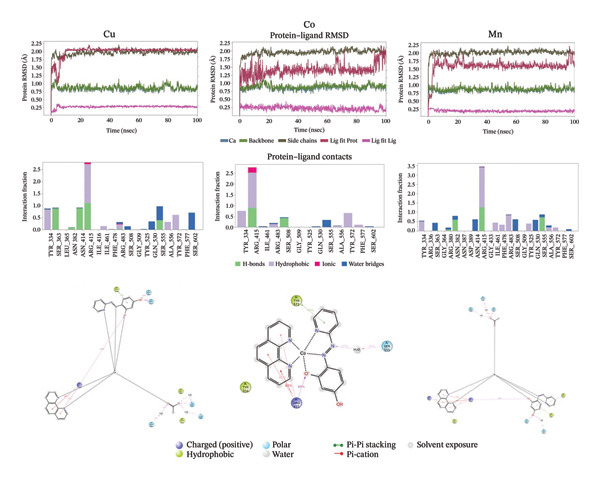
Molecular dynamics analysis (MD) of Cu, Co, and Mn complexes with Keap1 receptor.

RMSD analysis offers insights into the entropic role and inherent flexibility of the binding sites, as demonstrated by the Lig fit Prot and Lig fit Lig curves. In Mn/Cu complexes (Complex 1/3) with a charge of −2, ionic and water‐bridge bonds formed with the cationic regions (Lys/Arg) and acidic residues (Asp/Glu) tend to limit ligand extrusion, which is consistent with the consistently low or stable Lig fit Prot RMSD. The ligand RMSD for the Cu complex (Complex 3) within the binding pocket is shown by the red line, with a value of 2.25 Å, while the pink line indicates a ligand RMSD of 0.25 Å. Similarly, the Mn complex (Complex 1) displays comparable RMSD values, with ligand RMSD in the binding region remaining below 2.00 Å and a ligand RMSD of 0.25 Å. In the Co (−1) complex (Complex 2), hydrophobic and *π* interactions are dominant, resulting in a profile more sensitive to micropore changes. RMSD values are also low, often below 2.00 Å. RMSD fluctuations over 100 ns are illustrated in Figure 9.

Contact types are categorized into four main classes: H‐bonds, hydrophobic interactions, ionic interactions, and water bridges, represented by green, gray, pink, and blue bars in Figure 9. In the Cu and Mn (−2) complexes, ionic and water‐bridge contacts are more prominent and continuous, with a more consistent H‐bond network. Specifically, the Cu complex shows five hydrogen bonds involving SER363, ASN382, ASN414, ARG415, and SER555. For the Mn complex, ARG380, ASN382, ARG415, and SER555 form similar hydrogen bonds. In the Co (−1) complex, hydrophobic/*π* interactions contribute more significantly, leading to more fragmented interaction regions. Only two hydrogen bonds are observed with ARG415 and Ser508. Across all three complexes, ARG415 is the most frequently interacting residue, engaging in ionic, hydrophobic, and hydrogen bonds, as detailed in Table 9.

**TABLE 9:** Molecular interactions of Cu, Co, and Mn complexes with Keap1 receptor and xanthine oxidase receptor as a result of molecular dynamics analysis (MD).

	Keap‐1 receptor			Xanthine oxidase receptor		
	Complex 3 [Cu(PAR) (Phen)AcO]·H_2_O	Complex 2 [Co(PAR) (Phen) (H_2_O)]AcO·H_2_O	Complex 1 [Mn(PAR) (Phen)AcO]·H_2_O	Complex 3 [Cu(PAR) (Phen)AcO]·H_2_O	Complex 2 [Co(PAR) (Phen) (H_2_O)]AcO·H_2_O	Complex 1 [Mn(PAR) (Phen)AcO]·H_2_O
Water content	7870	8157	7875	30,846	31,995	30,839

Atoms	28,057	28,866	28,072	111,606	114,873	111,587

Total charge	0	0	0	0	0	0

Protein SSE	β ∼%46.9; heliks ∼%0	β ∼%47.2; heliks ∼%0	β ∼%46.5; heliks ∼%0	β ∼%21.17; heliks ∼%27.52	β ∼%21.27; heliks ∼%27.81	β ∼%21.18; heliks ∼%27.09

Ligand charge/formula	−2; C_25_H_19_CuN_5_O_4_	−1; C_23_H_16_CoN_5_O_2_	−2; C_25_H_19_MnN_5_O_4_	−2; C_25_H_19_CuN_5_O_4_	−2 C_23_H_15_CoN_5_O_2_	−1 C_25_H_20_MnN_5_O_4_

Num. of atoms	54 (total) 35 (heavy)	47 (total) 31 (heavy)	54 (total) 35 (heavy)	54 (total) 35 (heavy)	46 (total) 31 (heavy)	55 (total) 35 (heavy)

Ionic environment	Na^+^(29)Cl^−^ (22)	Na^+^(6)	Na^+^(29)Cl^−^ (22)	Na^+^(86)Cl^−^ (94)	Cl^−^ (8)	Na^+^(86)Cl^−^ (95)

Dominant contact types	Ionic + water‐bridge + H‐bond	Hidrofobik/*π* (π–π, π–katyon)	Ionic + water‐bridge + H‐bond	Ionic + water‐bridge + H‐bond	Ionic + water‐bridge + H‐bond	Ionic + water‐bridge + H‐bond + hydrophobic/*π* interactions

RMSD (Lig fit Prot)	2.25	2.00	2.00	2.4	1.6	2.8

Protein–ligand contacts (cont.)	High (continuous bands) ARG415,ASN414,SER363,TYR334	Medium (more fragmented) ARG415, TYR334, SER508,TYR572	High (continuous bands) ARG415, TYR334, SER363, ASN382, ASN414, SER555	High (continuous bands) GLU45,LYS422,GLN423, SER425, ARG426, LYS433,LYS1228	High (continuous bands) ASP360, LYS422, ARG426, LYS433, LYS1228	High (continuous bands) ASP360, LYS422, ARG426, LYS433, LYS1228

Protein–ligand contacts (H‐bonds)	SER363, ASN382, ASN414,ARG415, SER555	ARG415, SER508	ARG380, ASN382, ARG415, SER555	GLU45, LYS422, SER425,ARG426, LYS433,LYS1228	LYS422, ARG426, LYS433	ASP360, ARG426,LYS1228

Protein–ligand contacts (İonic‐int.)	ARG415, ARG483	ARG415	ARG415, ARG483	LYS422, LYS433, LYS1228	LYS422, ARG426, LYS433, LYS1228	LYS433, LYS1228

Protein–ligand contacts (hydrophobic‐int.)	TYR334,ARG415, ILE461, PHE478, ARG483, ALA556, TYR572	TYR334, ARG415, ARG483, ALA556, TYR572, PHE577	TYR334, ARG415, ILE461, PHE478, ARG483, TYR525, ALA556,TYR572	TRP336, LYS422, ALA424, ARG426,ALA1231	LEU147, TRP336, ALA338, LYS422, LEU1208, PRO1224, ALA1231	LEU147, TRP336, PHE337, ALA338, LYS422, LEU1208, ALA1231

Ligand–protein contacts	ARG415 (pi‐cation) ARG415 (H‐bond) TYR334 (pi‐pi stacking) SER363 (H‐bond) ASN414 (H‐bond) SER555 (H‐bond) GLN530 (H‐bond) SER602 (H‐bond)	ARG415 (pi‐cation) ARG415 (H‐bond) TYR572 (pi‐pi stacking) SER555 (H‐bond)	ARG415 (pi‐cation) ARG415 (H‐bond) ARG483 (pi‐cation) TYR572 (pi‐pi stacking) TYR334 (pi‐pi stacking) ASN382 (H‐bond) ASN414 (H‐bond) SER555 (H‐bond) GLN530 (H‐bond) SER508 (H‐bond)	LYS422 (pi‐cation) LYS422 (H‐bond) LYS433 (H‐bond) GLU45 (H‐bond) LYS1228 (H‐bond) SER425 (H‐bond) GLN423 (H‐bond)	LYS422 (pi‐cation) LYS422 (H‐bond) LYS1228 (salt b.) LYS433 (H‐bond) ASP360 (H‐bond) ARG426 (H‐bond)	LYS1228 (H‐bond) LYS1228 (pi‐cation) ARG426 (H‐bond) LYS422 (H‐bond) LYS433 (H‐bond) ASP360 (H‐bond)

In the Cu complex, Arg415 participated in pi‐cation interactions with the 1,10‐phenanthroline (phen) part of the ligand and formed hydrogen bonds with the O atom in the 4‐(2′‐pyridylazo) resorcinol group. TYR334 engaged in pi‐pi stacking with the 4‐(2′‐pyridylazo) resorcinol moiety, while SER363 and ASN414 established hydrogen bonds with the OH group. Additionally, SER555, SER602, and GLN530 contributed by creating water‐mediated polar interactions with the OAc group linked to the ligand.

Since the Mn complex structure has a similar molecular formula and charge to the Cu complex structure, similar interactions were also valid for the Mn complex. For the Mn complex structure, ARG415 and ARG483 showed pi‐cation interaction with the 1,10‐phenanthroline (phen) moiety of the ligand. While TYR334 and TYR525 exhibited pi‐pi cation interactions with the 4‐(2′‐pyridylazo) resorcinol moiety of the ligand, ASN382 and ASN414 made hydrogen‐bond interactions with the OH terminus. The residues interacting with the AcO moiety of the ligand are SER508, GLN530, and SER555.

In the water‐dependent MD analysis of the Co complex structure, the molecule’s charge and its binding to water instead of the acetyl group led to a different interaction profile. ARG 415 engaged in both pi‐cation and hydrogen bonding with the PAR and phen moieties of the ligand. TYR572 showed pi‐pi stacking interactions with the PAR moiety, while the polar SER555 formed water‐mediated hydrogen bonds. In this receptor environment, the binding stability and resistance to pocket displacement follow the order Cu ≈ Mn ≥ Co. The ionic and water‐bridged networks in the Cu/Mn complexes are more continuous than the hydrophobic/*π* network observed in the Co complex. All these interactions are detailed in Table 9 and Figure 9.

### 3.11. MD Results of XO Receptor Interactions

Root mean square deviation (RMSD) analyses of all three complexes with the XO receptor were conducted to compare the stability of the proteins and ligands during the simulation. The RMSD plots, aligned on the protein backbone, indicate the systems’ stability. For the Cu complex (Complex 3–XO complex), the Lig fit Prot RMSD stabilized around ∼2.4 Å with minimal fluctuations throughout the simulation. The ligand RMSD values remained low and consistent with the protein RMSD, staying stable within the binding site. In the Co complex (Complex 2), the main chain RMSD, shown in light green, stabilized near ∼1.6 Å, while the Lig fit Prot curve stayed below 1.6 Å, indicating continuous ligand presence in the binding pocket. For the Mn complex, the Lig fit Prot RMSD stabilized between 2.0 and 2.8 Å; the ligand RMSD data suggest strong binding and overall system stability.

In most simulations of the Cu complex, hydrogen bonds and ionic interactions played a major role. Long‐lasting contacts (∼70%–90%) were especially noted with residues LYS422, SER425, and CYS1228. LYS422 exhibited various interactions, including strong hydrogen bonds with the O atom of the PAR ligand and pi‐cation interactions with the phen group. The GLU45 residue interacted with the PAR hydroxyl group through water‐mediated contact. LYS433 also formed hydrogen bonds with the negatively charged O atom in PAR. Additionally, GLN423, SER425, and LYS1228 engaged in hydrogen bonding with the acetyl group. Residues LYS422, LYS433, and LYS1228 contributed to ionic contacts through their interactions.

The Co complex exhibited a different binding profile because it was designed without an acetyl group binding site, and the system’s ionic capacities were not equivalent to those of the Cu and Mn systems. Only 8 Cl^−^ ions were added to neutralize the Co complex. In this case, the primary interactions are ionic, with residues LYS422, ARG426, LYS433, and LYS1228 playing key roles. Besides ionic interactions, LYS422, ARG426, and LYS433 also form hydrogen bonds with the complex. Throughout the 100 ns simulation, residues LYS422, LYS433, and LYS1228 consistently interacted with the complex. Specifically, LYS422, LYS1228, and ARG426 strongly interacted with the negatively charged oxygen atom in the PAR group, while LYS433 and ASP360 interacted with the other negatively charged oxygen atom. Additionally, PRO1224 and ALA1231 contributed to the complex’s structural stability through hydrophobic interactions.

**FIGURE 10:**
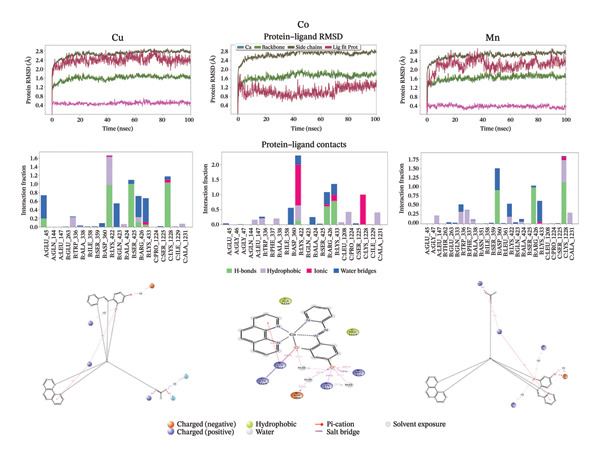
Molecular dynamics analysis (MD) of Cu, Co, and Mn complexes with xanthine oxidase receptor (XO).

The negatively charged Mn complex was neutralized by an equivalent number of ions from the Cu complex. The main interactions observed were hydrogen bonds and hydrophobic contacts. Specifically, hydrogen bonds were identified with residues ASP360, ARG426, and LYS1228, while ionic interactions involved LYS433 and LYS1228. Hydrophobic interactions were seen with residues LEU147, TRP336, PHE337, ALA338, LYS422, ALA424, LYS1228, and ALA1231. Notably, the interactions between ASP360, ARG426, and LYS1228 with the complex are ongoing. LYS422 and LYS433 interacted via hydrogen bonds with the hydroxyl groups of the PAR molecule, and ASP360 interacted with an alternate hydroxyl. LYS1228 formed both pi‐cation and ionic interactions with the PAR group and hydrogen bonds with the acetyl group. ARG426 interacted only with the acetyl group, and no residues engaged with the phen group. All these interactions are illustrated in Figure 10 and summarized in Table 9.

The Cu and Co complexes (net −2) create stronger ionic and water‐bridged networks, whereas the Mn complex (net −1) shows more hydrophobic and π‐character. This difference is evident in how well the ligand maintains its position within the pocket (Lig fit Prot RMSD) and in the consistency of the contact bands.

## 4. Conclusions

In this study, new mixed‐ligand metal(II) complexes containing the azo ligand PAR and 1,10‐phenanthroline were synthesized. The obtained complexes were characterized by elemental analysis, molar conductance measurements, thermal analysis, magnetic susceptibility, and IR spectroscopy. The results indicate that the tridentate azo ligand coordinates to the metal(II) ions through the phenolic oxygen, azo nitrogen, and pyridylazo nitrogen atoms, while 1,10‐phenanthroline binds through its nitrogen atoms, completing the coordination environment. The FRAP and CUPRAC assays demonstrated that metal coordination significantly altered the antioxidant properties of the ligands, that the reducing capacities of the complexes varied depending on the redox system employed, and that Complex 1 exhibited the highest activity in the FRAP assay, whereas Complex 2 showed the highest activity in the CUPRAC assay. Similarly, all complexes exhibited an electrochemical response, and the oxidation potentials followed the order Complex 1 (Mn) < Complex 3 (Cu) < Complex 2 (Co), indicating that the metal ions influence the electronic environment of the ligand to different extents. The diffusion‐controlled nature of the electrochemical processes and the redox behavior of all complexes suggest that these structures may be promising candidates for electrochemical sensor applications. The integration of quantum chemical calculations with molecular docking and molecular dynamics simulations provided a comprehensive understanding of the structure–activity relationships of the studied complexes. HOMO‐LUMO energy gaps and MEP charge distributions demonstrated the reactive nature and potential binding hotspots of the complexes, while docking and MD simulations confirmed their stable binding within the active sites of XO and Keap1. These findings indicate the promising bioactive potential of the investigated complexes. Furthermore, although related phenanthroline/PAR‐based metal complexes have been reported previously, the Mn(II), Co(II), and Cu(II) complexes synthesized in this study are new. The combined experimental and theoretical investigations, including antioxidant, electrochemical, molecular docking, molecular dynamics, and DFT studies, provide new insights into the structure–property and structure–activity relationships of this class of coordination compounds and broaden the current understanding of their potential functional and biological applications.

## Funding

No funding was received for this manuscript.

## Conflicts of Interest

The authors declare no conflicts of interest.

## Data Availability

The data that support the findings of this study are available upon request from the corresponding author. The data are not publicly available due to privacy or ethical restrictions.
